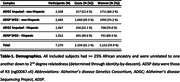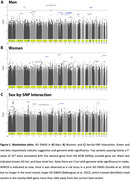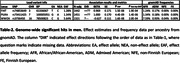# Sex‐stratified GWAS for Alzheimer’s Disease Reveals African Ancestry Risk variants in Men

**DOI:** 10.1002/alz.087086

**Published:** 2025-01-03

**Authors:** Michael E. Belloy, Danielle M. Reid, Yann Le Guen, Valerio Napolioni, Michael D Greicius

**Affiliations:** ^1^ Washington University in Saint Louis, Saint Louis, MO USA; ^2^ Stanford University, School of Medicine, Stanford, CA USA; ^3^ University of Camerino, Camerino Italy; ^4^ Department of Neurology and Neurological Sciences, Stanford University, Stanford, CA USA

## Abstract

**Background:**

Several studies have indicated sex‐specific genetic risk for Alzheimer’s disease (AD), but these were centered on non‐Hispanic White individuals of European ancestry. We sought to identify sex‐specific genetic variants for AD in non‐Hispanic and Hispanic subjects of admixed African ancestry.

**Method:**

Participants were ages 60+, of African ancestry (≥25%), and diagnosed as cases or controls. Genetic data were available from SNP arrays imputed to TOPMed or whole genome sequencing (WGS). Genome‐wide association studies (GWAS) were performed per data type, sex, and Hispanic/non‐Hispanic status (**Table‐1**), requiring genotyping rate ≥80% and minor allele frequency ≥1%, followed by meta‐analysis (Plink v2.0; GWAMA v2.2.2). A sex‐by‐SNP interaction model was also evaluated. GWAS performed multiple linear regression on an AD‐age score (Le Guen & Belloy et al. 2021; **Figure‐1**), adjusting for APOE*4/APOE*2 dosage, the first five genetic principal components (PC‐AiR; GENESIS; R v3.6), and array/sequencing center. Variants were filtered to be covered on at least half of the individuals per stratum.

**Result:**

We found 3 genome‐wide significant loci in men (**Figure‐2**) with lead variants 1) rs74853649 ∼85kb downstream of *FHIT*, 2) rs73411425 in the *PILRA/ZCWPW1/NYAP1* AD risk locus, and 3) rs3736450 intronic on *WWOX*. Despite that rs73411425 is within a known locus, the variant is novel. Notably, all three variants showed significant sex‐by‐SNP interactions and were specific to African rather than European ancestry (**Table‐2**). Further, in men, we also observed a suggestive association intronic on *RBFOX1*, which was previously implicated as a risk gene harboring variants associated with higher amyloid burden in brain (Raghavan et al. 2020), with strongest effect sizes in African‐American individuals (those samples were independent of the current data).

**Conclusion:**

The study is an initial exploration of sex‐specific genetic effects in African ancestry individuals and warrants replication in larger data. Importantly, we show the relevance of expanding beyond non‐Hispanic white samples for sex dimorphism research, identifying male‐specific risk variants that have elevated frequencies in African ancestry, while being rare in other ancestries. In ongoing work, we are performing cross‐ancestry meta‐analyses of sex‐stratified GWAS followed by sex‐stratified omics integration to increase power to uncover sex‐specific genes relevant to AD.